# Evaluation of integrated disease surveillance and response (IDSR) and early warning and response network (EWARN) in South Sudan 2021

**DOI:** 10.11604/pamj.supp.2022.42.1.33780

**Published:** 2022-06-09

**Authors:** John Rumunu, Joseph Francis Wamala, Robert Sakaya, Sheila Baya Konga, Alice Lado Igale, Abraham Abenego Adut, Scopas Korsuk Lonyik, Robert Martin Lasu, Rose Dagama Kaya, Guyo Guracha, Peter Nsubuga, Fabian Ndenzako, Ambrose Otau Talisuna

**Affiliations:** 1 Doctoral Program in Global Health, Humanitarian Aid and Disaster Medicine, Universita Del Pemonte Orientale and Vrije University Brussel, Juba, South Sudan; 2 World Health Organization Country Office for South Sudan, Juba, South Sudan; 3 Independent Consultant, Juba, South Sudan,; 4 Global Public Health Solutions, Juba, South Sudan; 5 World Health Organization Regional Office for Africa, Brazzaville, Republic of Congo

**Keywords:** Disease surveillance, response, detect, priority diseases, feedback

## Abstract

**Introduction:**

South Sudan has been implementing the Integrated Disease Surveillance and Response (IDSR) strategy since 2006, along with Early Warning and Alert Response and Network (EWARN). The IDSR/EWARN stakeholders commissioned an independent evaluation to establish performance at national, state, county, health facility, and community levels in the first half of 2021.

**Methods:**

the evaluation was conducted between June and September 2021 (during the COVID-19 pandemic) and was based on the World Health Organization (WHO) protocols for monitoring and evaluating communicable disease surveillance and response systems and the guidelines for evaluating EWARN.

**Results:**

integrated disease surveillance and response/early warning and alert response and network indicator data showed improving timeliness and completeness from the beginning of 2021 to week 16 and then a slight depression of timeliness by week 32, while completeness remained high. Event-based surveillance was active at the beginning of 2021 and in week 32. However, there was inadequate sample collection to investigate acute watery diarrhea, bloody diarrhea, and acute jaundice syndrome alerts. Respondents in all cadres had substantial experience working in IDSR/EWARN. All respondents performed the various IDSR/EWARN tasks and duties as expected, but needed more resources and training.

**Conclusion:**

while IDSR/EWARN is performing relatively well, confirmation of priority diseases by the laboratories needs to be strengthened. Health facilities need more regular supervision from the higher levels. Community health workers need more training on IDSR/EWARN. The whole IDSR/EWARN system needs more resources, particularly for communication and transport and to confirm priority diseases. Staff at all levels requested more training in IDSR/EWARN.

## Introduction

Beginning in 1998, the Integrated Disease Surveillance and Response (IDSR) has remained the overarching strategy for strengthening and building robust national disease surveillance systems and is the vehicle for attaining the international health regulations (IHR 2005) core capacity requirements in the World Health Organization (WHO) Africa Region [1-3]. South Sudan has been implementing the IDSR strategy since 2006 [2]. South Sudan has experienced several outbreaks over the years and is part of the yellow fever and meningitis belts [4-8]. Integrated disease surveillance and response aims to establish a national disease surveillance system with capacities to detect, report, confirm and effectively respond to high-priority communicable and non-communicable diseases and other events of public health importance. Given the humanitarian context of the country, South Sudan implemented IDSR alongside the Early Warning Alert and Response Network (EWARN) as an adjunct system that supports surveillance and response needs in locations where IDSR was underperforming due to security constraints. By design, EWARN and similar systems focus on rapid notification of epidemic-prone and other emergent public health diseases [9].

Public health surveillance and response systems require regular evaluations to ensure that they are still aligned with their objectives. In recent years, South Sudan and its partners have invested in IDSR and EWARN [10]. There was a comprehensive mid-term evaluation of the IDSR strategy in South Sudan in 2011 [10]. South Sudan adopted and is using the third edition of the IDSR Technical Guidelines in 2019 [11,12]. In late 2020, the stakeholders commissioned an evaluation to establish the status and performance of IDSR/EWARN capacities at the national, state, county, health facility, and community level. The assessment was to determine the surveillance capacities. The capabilities to detect, report, investigate, analyze, prepare, respond, and provide feedback by surveillance and laboratory focal points, rapid response teams, emergency preparedness and response teams, and other stakeholders at all levels were evaluated. The assessment focused on the first half of 2021. The worldwide COVID-19 pandemic coincided with the evaluation period and reduced the planned size [13]. The final objectives of the evaluation were to describe the IDSR and EWARN systems and how they relate and operate in South Sudan and assess the effectiveness and usefulness of IDSR and EWARN to detect, confirm, and respond to diseases, outbreaks, and events of public health importance. We present the approach, results, and recommendations from the IDSR/EWARN evaluation.

## Methods

**Approach:** the evaluation was based on the WHO protocol for monitoring and evaluating communicable disease surveillance and response systems and the guidelines for evaluating early warning alert and response networks [14-16]. The evaluation occurred between June and September 2021.

**Evaluation team:** the evaluation was conducted by an independent team comprising a team leader based in Atlanta and a team member based in Juba. They were supported by an IT team based in Atlanta. Because of funding and the COVID-19 pandemic, the plans for the local component of the evaluation were scaled back. The evaluation team reported to the Technical Officer for Emergencies in the WHO Juba Office and the Director General, Preventive Health Services, Ministry of Health who provided technical guidance.

### Process

Obtaining background information: the evaluation team identified and obtained all critical documents for providing background information and operational context of IDSR and EWARN. These documents were obtained from the Ministry of Health and WHO and were provided in a shareable folder online.

### Site selection

Selection of states: for purposes of this evaluation, the country was divided into three Regions- Equatoria, Bahr el Ghazal, and Upper Nile. The assessment was conducted in one State in each of the three Regions of South Sudan: Equatoria - Eastern Equatoria State, Bahr el Ghazal - Northern Bahr el Ghazal State, Upper Nile - Upper Nile State. The final site selection depended on the availability of respondents given the COVID-19 pandemic travel and other restrictions. There were also team size restrictions based on the pandemic and funding considerations.

**Tool adaptation and piloting:** data collection tools were adapted to the context of the South Sudan health system and IDSR/EWARN systems. After adaptation, they were piloted to ensure they were comprehensive, appropriate, readable, relevant, and understandable. After the piloting, the tools were updated based on the feedback and finalized for conducting the evaluation. The data were presented in three parts: i) Indicator and event-based surveillance data; ii) quantitative data from the evaluation; iii) surveillance attributes of the IDSR/EWARN system. Conclusions were made from each of the analysis parts, and recommendations were made to the stakeholders.

**Ethics approval and consent to participate:** administrative clearance for publication of this paper was provided by the Ministry of Health of South Sudan and WHO (WHO e-Pub no: ePub-IP-00332847-EC).

## Results

Indicator data: at the beginning of 2021, IDSR timeliness ranged from 37% to 95%, most states were below the target (80%), and the average was 66%. Integrated disease surveillance and response completeness ranged from 50% to 100%, most states were above the target, and the average was 87%. During the same period, county-level IDSR timeliness was 34 (42%), while completeness was 53 (66%) [17]. In weeks 15 and 16, both IDSR timeliness and completeness had markedly improved from where they were at the beginning of 2021. The average for timeliness was 91%, up from 66% and for completeness, it was 92%. Country-level completeness in reporting improved from 53 (66%) in week 1 of 2021 to 62 (78%) in week 16 of 2021 [18]. IDSR timeliness in week 32 was 84%, which improved from week 31 but a drop from week 16. Average completeness in week 32 was 90% which was similar to week 16. Country-level timeliness in week 32, 2021 was 60 (75%), representing a decline from week 16, 2021 [19].

**Event-based surveillance:** a snapshot of event-based surveillance from weeks 1 and 32 showed multiple alerts from several states. The commonest alerts were acute watery diarrhoea (AWD) and malaria, followed by bloody diarrhoea (BD). The number of states reporting AWD alerts increased from five in week 1, 2021 to nine in week 32, 2021, but the number of states collecting samples to investigate AWD alerts reduced from 6 (86%) to no samples collected to investigate AWD alerts in week 32, 2021. The number of states reporting BD alerts increased from five to seven from week 1 to week 32, 2021, but no samples were collected to investigate the cases reported during the period [17,19].

**Quantitative data from the questionnaires:** a total of 33 participants responded to the various survey questionnaires either in person or via downloadable links. They were 10 surveillance officers, 10 community health workers, five health facility-based respondents, four senior-level county and central personnel, two laboratory-based respondents, and two data managers.

**Surveillance officers:** ten surveillance officers responded. Their locations were five from Northern Bahr el Ghazal State, two from Upper Nile State, two from Eastern Equatoria State, and one from Western Equatoria State. The surveillance officers had experience with IDSR/EWARN ranging from 3 to 156 months, five had an experience of 18 months or more, and were either state or country surveillance officers. Their primary responsibilities were data review, compilation/aggregation, data entry, and alert/outbreak investigation.

**Reporting sites:** the surveillance offices had between 11 and 27 sites reporting to them. Seven of the 10 surveillance officers reported difficulty accessing some of their reporting sites. The commonest reasons for lack of access to reporting sites were the lack of transport and communication. A total of 6 of the 10 surveillance officers reported that all their reporting sites functioned without disruptions or closures in the previous 3 months.

**Supervision:** the surveillance officers used various methods to supervise their reporting sites. The commonest method was facility visits, followed by telephone calls and training workshops. When asked to report on the percentage of reporting sites that the surveillance officers had visited in the previous 4 weeks, the majority, 6/9, reported that they had visited all the reporting sites. The commonest reasons for visiting the sites were regular supervision (7/9), collecting reporting forms (7/9), and providing feedback (6/9).

**Review of the weekly reporting form:** all surveillance officers reported that they reviewed the weekly reporting form from their reporting sites. The most typical reason for reviewing the form was to look for missing data.

**Feedback to reporting sites:** the commonest frequency of providing feedback to reporting sites was reported to be weekly. Primarily by visiting the site or telephone contact. All surveillance officers reported that they conduct meetings with their reporting sites and the commonest frequency was monthly. Most meetings occurred in person. The topics that were discussed at the meeting ranged from strengthening practices discussed in all meetings to outbreak investigation and response, which was discussed in 62.5% of meetings.

**Meetings with Ministry of Health/WHO Central level staff:** a total of 5/9 surveillance officers reported that they had regular meetings with the Ministry of Health or WHO Central level staff. Those who had meetings reported that they were held monthly and mostly in person. The reasons given for the lack of meetings are primarily a lack of funds and other resources for transportation. Those who had meetings reported that progress since the last meeting was the most discussed topic.

**Training in IDSR/EWARN:** a total of 4/9 reported that they had received training in IDSR/EWARN in the past 12 months. Those who had received that training indicated that WHO and the Ministry of Health had provided it and case definitions, outbreak investigation, data analysis, specimen collection, form completion, case management, and preparedness.

**Notification of IDSR/EWARN alerts:** the surveillance officers reported that they were most notified about alerts through the bulletins and their reporting channels. Six out of the seven who responded to the question indicated that they maintained an outbreak log or register. All responded that they were involved in alert verification; however, only 5/7 reported being involved in an outbreak investigation.

**Resources for alert and outbreak investigation:** all surveillance officers reported alert forms, registers, and specimen collection tools. The least resource they had was transportation. Among those who responded, all had tally sheets and weekly reporting forms. Less than half had transportation to aid in weekly reporting.

**Changes to IDSR/EWARN:** surveillance officers indicated that the commonest change to IDSR/EWARN since they had started working on it was adding or removing reporting sites. A total of 4/9 surveillance officers reported that new sites had been added to supervise in the previous 6 months.

**Integrated disease surveillance and response bulletins:** all surveillance officers reported that they received the weekly bulletins and found them helpful in various ways, especially in monitoring health trends; 4/7 reported that they believe the surveillance bulletins could be modified to make them more useful by enabling them to send them to reporting sites. The surveillance officers indicated that they share the bulletins with various stakeholders (e.g. other surveillance officers, IDSR/EWARN staff at health facilities, and reporting sites) primarily by email.

**Challenges with implementing IDSR/EWARN:** the surveillance officers reported that they had various challenges in implementing IDSR/EWARN. The commonest was the lack of sufficient training, the lack of funds, and the lack of communication; they also mentioned a lack of commitment from higher authorities (5/7).

**Health facilities:** responses were obtained from five health facilities from Makal, Lopa/Lafon, Juba, Maban, and Magwi counties. Four of the five were PHCCs the other was a hospital. The populations in their catchment areas ranged from 280,00 to 648,441. Three of the five belonged to the government, and the other two were nongovernment. The respondents had spent a minimum of 9 months working on IDSR/EWARN to a maximum of 141 months. The only source of interruption in their work was staff absenteeism reported by one respondent, and they spend a median of 8 hours per week working on IDSR/EWARN duties. Other reporting tasks as reported by 3/5 included malaria screening and monthly treatment summaries. Their primary responsibilities in IDSR/EWARN were mainly cased detection, alert, and outbreak investigation.

**Supervision:** respondents who answered the question reported that they received supervision from a higher level, mostly weekly, as indicated below. The supervision was mainly through facility visits. Supervisors came to strengthen practices, review progress, and collect IDSR/EWARN data.

**Feedback:** respondents reported that they primarily received feedback weekly on the data collected on IDSR/EWARN, primarily by facility visits.

**Meetings with local staff:** strengthening practices was the most frequently mentioned topic that is discussed at meetings with local staff at the health facility level. Three out of four respondents reported that they trained local staff sporadically. The training at the health facilities comprised case definitions, data analysis, specimen collection, and form completion primarily.

**Immediate reporting:** facility respondents were asked to indicate conditions that required immediate reporting. Acute bloody diarrhoea was not identified as a condition that required immediate reporting by half of the respondents. The respondents were also split on malaria.

**Alert notification and outbreak investigation:** one of the four health facility respondents reported being involved in outbreak investigations. Three of the four respondents reported they had an outbreak log; however, two only reported a method to update the cases in their registers with laboratory results. Not all the health facility respondents had all the resources needed for outbreak investigation and response. Two of the three had alert forms and registers, and specimen collection tools.

**Feedback:** all health facility respondents reported that they had not received any weekly bulletins but indicated that the bulletins would be useful if they were receiving them.

**Utility of IDSR:** all respondents indicated that IDSR/EWARN is useful for monitoring health trends, detecting early outbreaks, and sharing information with partners. They all had used IDSR to respond to outbreaks in their catchment area.

**Challenges in IDSR/EWARN:** all those who responded indicated that insufficient training was the most challenging part of IDSR/EWARN.

**Community health workers:** ten community health workers responded to the community health worker questionnaire. They were primarily based at the Payam level. They indicated that they mainly visited their communities weekly. Six out of 10 respondents stated that they were familiar with IDSR/EWARN.

**Frequency of visiting communities by community health workers:** a total of 7 of 9 respondents indicated locations within their catchment area that they could not visit. The commonest reason was lack of transportation, followed by communication and security. The commonest conditions that the community health workers identified were diarrhoea, malaria, and malnutrition.

**Training:** five out of nine community health workers could provide information about the most recent training they received. The training lasted 2 days and was provided by WHO and World Vision. All community health workers mentioned case definitions, diagnosis, and specimen collection. All respondents identified AFP as an immediately notifiable condition.

**Alert and outbreak investigation:** four out of 10 respondents were involved in or participated in an alert verification or investigation. Their roles were mainly in sample collection.

**Supervision:** respondents received supervision from health authorities above them every month mostly. However, 2/9 reported that they had rarely or never received supervision. Most supervision was provided on an individual basis.

**Challenges faced by community health workers:** communication problems and not enough training were identified as the main challenges community health workers face in their work.

**Laboratories:** two public health laboratories were surveyed in the evaluation, one in Upper Nile State (Makal County) and the other in Easter Equatoria State (Ikwoto County). One was at a hospital the other was at a Primary Health Care Corporation (PHCC). Both respondents reported that their primary responsibilities included laboratory sample collection, conducting laboratory tests, laboratory quality control, and supervision. Both respondents were laboratory technicians. Both laboratories were reportedly open every day of the week from 8am to 5pm. One of the laboratories reportedly could accept samples after hours, and both conducted routine laboratory tests.

**Reporting, notification:** both laboratories did not have standardized reporting forms or standardized forms to transfer specimens to other laboratories and reported their results using cellphones for immediately notifiable conditions. They both did not have a written policy for rapid notification of outbreak specimens (e.g. measles and cholera). Both laboratories reportedly did not have specimen logbooks.

**Resources:** the hospital laboratory had more resources (i.e. refrigerator, centrifuge, balance scale, generator) than the PHCC laboratory, and they were monitored and calibrated. None of the laboratories had an incubator. Both laboratories had adequate stocks of reagents for the tests they conducted.

**Training:** both respondents reported that none of their staff had been trained and certified in shipping laboratory specimens.

**Central and county level respondents:** four senior-level respondents at the central and county level were surveyed, a disease surveillance officer, an EPI manager, a county health director, and an EPI officer. They were located at the State Ministry of Health or the Ministry of Health and had spent between 5 and 240 months in IDSR/EWARN. Their primary responsibilities in IDSR/EWARN are as indicated in the chart below. They also had non-IDSR/EWARN responsibilities-case management, administrative duties, and emergency response.

**Review of IDSR data:** the respondents reported that they all reviewed the IDSR data to identify discrepancies, missing data, significant variations in numbers, and unusual or new diseases.

**Feedback to surveillance or health staff:** when asked how often they gave feedback to their direct reports, the majority gave daily and weekly feedback. They gave the feedback using various methods, including email, phone calls, and facility calls.

Supervision, meetings with direct reports: three out of four respondents indicated that they supervised health staff on data collected weekly using various methods, including email, cellphone, facility visits, and training. Half of them indicated that they held regular meetings with surveillance officers, those who did not blame the lack of funds for holding these meetings. The topics that were discussed at the meetings were primarily to strengthen practices.

**Training of surveillance staff:** respondents were asked how often they or their agency/organization provided training to surveillance staff reporting to them, and it mainly was sporadically dependent on time and funding.

**Alert notification:** respondents indicated that they received IDSR/EWARN notifications from health facility staff, surveillance staff, reporting channels, and the community primarily. Three of the four respondents indicated that they maintained an outbreak log at their level. The respondents indicated that their IDSR/EWARN systems were linked with other systems (e.g. alpha-fetoprotein (AFP), measles, malaria).

**Resources:** respondents indicated that they had several types of resources for alert and outbreak investigations, mostly specimen collection tools and equipment.

**Integrated disease surveillance and response/early warning alert and response network bulletins:** all respondents found the IDSR/EWARN helpful in monitoring health trends in their areas, assisting in community campaigns, and sharing findings with stakeholders. Other reasons included monitoring timeliness and completeness and providing feedback about the health situation in their countries. The bulletins were shared with surveillance officers, health facilities, laboratories, other partners, and non-governmental organizations via email, verbal summaries, and paper copies.

**Changes to IDSR/EWARN requirements, funding:** respondents indicated that IDSR/EWARN had expanded in the country since it was implemented. Two out of the four respondents indicated that they had been changes in IDSR/EWARN in the last 2 years. In terms of funding, the respondents indicated that they were unsure of financing IDSR/EWARN for the next 12 months.

**Challenges in IDSR/EWARN:** respondents identified several challenging parts in implementing IDSR/EWARN, primarily insufficient training and a lack of funding. Other challenges were a lack of communication and a lack of response to outbreak alerts.

**Data managers:** two data managers were surveyed as part of the evaluation. They were both based in Juba in Central Equatoria State and had spent 8 years working on IDSR/EWARN. Their primary responsibilities were data review, data analysis and interpretation, and the production of weekly bulletins. They also worked on non-IDSR/EWARN data. They reported that IDSR/EWARN data were entered by health facility in-charges, county and state surveillance officers, and monitoring and evaluation officers. They found IDSR/EWARN useful for early outbreak detection, monitoring health trends, and sharing information with partners.

**Data entry and cleaning:** data managers identified unusual or unexpected data by running the data through MS Excel, and if necessary, calling the data source to check the data entry. Lately submitted data are part of the calculation for completeness, but they reportedly advise the data sources to send their subsequent reports early. Missing data are also reportedly sent back to the source for re-entry.

**Data storage and backup:** respondents reported that data are stored locally and backed up by weekly downloads on different external drives or the cloud.

**Data management, analysis:** respondents reported that data are analyzed based on demands, either daily, weekly, or monthly. The data managers also analyze alpha-fetoprotein, Guinea Worm, and the other IDSR/EWARN data. They analyze the data using EWARS, MS Excel Kobo tool, or mapping software and share the results with surveillance officers and IDSR/EWARN stakeholders in WHO and the Ministry of Health for decision-making.

**Challenges in IDSR/EWARN:** the data managers reported challenges. They indicated that they would like to receive training on GIS, SPSS, and different data analysis tools. They suggested that IDSR/EWARN could be strengthened with quarterly training for all national and state data managers.

**Suggestion to improve IDSR/EWARN:** the survey respondents at all levels had various suggestions to improve IDSR/EWARN. The respondents needed more IDSR training, simulations, and supervision at all levels in addition to ample resources, e.g. funding, transporation and the EWARs and ODK phones for communication of surveillance information.

**Summary of surveillance attributes:** the IDSR/EWARN system was rated simple, flexible, medium to high data quality, medium to high acceptability, and medium representativeness (Table 1, Table 1 (suite)). Stability was rated high overall, and usefulness was rated high. Laboratory-based attributes, simplicity was rated high, data quality medium, and timeliness was medium to high, while stability was low (Table 2).

**Table 1:** surveillance attributes of the IDSR/EWARN in South Sudan 2021

Attribute and indicator	Assessed/not assessed	Evaluator rating Low/medium/high)
**Simplicity**		
IDSR/EWARN integration with other systems	Yes	High
Method to collect, manage, enter, analyze and disseminate data	Yes	High
Time spent on maintaining the system		
Amount and type of data collected for each priority disease (e.g. demographics, exposure information, etc)		
System training	Yes	Low
**Flexibility**		
Process to add/remove health units/partners	Yes	Medium
**Retrospective review of how system responded to a new demand such as**		
Emerging health events	Yes	High
Changes in case definitions	Yes	High
Variations in funding	Yes	High
**Data quality**		
Quality control practices		
Critical discussion of data and reports with partners	Yes	Medium
Use of standardized tools and forms	Yes	Medium
Staff who can correctly identify immediately notifiable diseases	Yes	High
Staff who accurately provide case definitions	Yes	High
Staff who accurately provide alert thresholds	Yes	High
Staff who can correctly explain the alert notification procedure	Yes	High
**Training**		
Current surveillance officers trained in IDSR/EWARN	Yes	Medium
Current IDSR/EWARN health facility staff trained in EWARN	Yes	Low
New IDSR/EWARN health facility staff (hired within the past 6 months) trained in EWARN		
New IDSR/EWARN partners/reporting sources (added within the past 6 months) trained in IDSR/EWARN		
Length of trainings (initial and refresher)	Yes	Medium
Most common/primary training topics	Yes	High
Primary training facilitators		
**Supervision and feedback**		
Health facilities which received feedback in previous 4 weeks; in previous 8 weeks	Yes	Medium
Health facilities which received supervisory visits in previous 4 weeks; in previous 8 weeks	Yes	High

**Table 1(suite):** surveillance attributes of the IDSR/EWARN in South Sudan 2021

Attribute and indicator	Assessed/not assessed	Evaluator rating (low/medium/high)
**Acceptability**		
Barriers to reporting		
Organization/agency/staff willingness to participate		
Perceived strengths and weaknesses of the system	Yes	Medium
Support and feedback to IDSR/EWARN staff	Yes	High
Regular meetings to review EWARN (strengthen practices, discuss progress, feedback, etc)	Yes	High
Internal review of the data		
Responsiveness of the system to suggestions or comments		
**Representativeness**		
Groups or subgroups not covered by or included in the system	Yes	Medium
Systematic exclusion or barriers to health care access		
**Stability**		
Functioning tools/equipment and resources for weekly surveillance and outbreak detection and response	Yes	High
Interruptions to reporting and impact on the system	Yes	Low
Costs involved to maintain the system		
Staff turnover		
Time in current position and EWARN-related activities	Yes	High
Uninterrupted weeks with functioning health facilities in the last 6 months	Yes	High
**Usefulness**		
Perceived usefulness of IDSR/EWARN data and bulletins	Yes	High
Ppublic health action (e.g., control measures implemented) based on data from EWARN	Yes	High
System's ability to meet its objectives	Yes	High
System's ability to help improve clinical, behavioral, social, policy or environmental practices		

**Table 2:** laboratory attributes of the IDSR/EWARN in South Sudan 2021

Attribute and indicator	Assessed/not assessed	Evaluator rating (low/medium/high)
**Simplicity**		
Priority conditions that can be laboratory-confirmed	Yes	Medium
Method for reporting results of immediately notifiable conditions	Yes	High
**Data quality**		
Use of standardized forms	Yes	Low
Legibility of laboratory registers		
Completeness of laboratory registers		
Diagnostic tests for which standard operating procedures are available	Yes	High
Diagnostic tests for which quality control is performed	Yes	Medium
**Specimen collection**		
Specimens received with a label, with a unique identifier		
Specimens received with adequate material for testing		
Specimens received in the recommended container, including packaging and temperature	Yes	Medium
Specimens received with associated specimen form		
Specimens with date and place of specimen collection on the form		
Specimens with all other data entries on the form completed		
Specimens with receipt time at laboratory recorded	Yes	High
**Timeliness**		
Samples expected to be analyzed within 24 hours, within 48 hours	Yes	High
Time from specimen arrival at the laboratory to results from the referral laboratory		
Time from specimen collection to arrival in the laboratory	Yes	High
Time from specimen arrival at the laboratory to testing	Yes	High
Time from testing until result reported to the collection site	Yes	Medium
Time from specimen collection until results reported.	Yes	Medium
**Stability**		
Staff reporting resources for specimen storage and diagnostic testing	Yes	Low

## Discussion

The independent IDSR/EWARN evaluation revealed several findings. Indicator data show improving performance in terms of timeliness and completeness of IDSR data from the beginning of 2021 to week 16 and then a slight depression of timeliness by week 32, while completeness remained high. Event-based surveillance was active at the beginning of the year and in week 32. There was inadequate sample collection to investigate acute watery diarrhoea, bloody diarrhoea, and acute jaundice syndrome alerts. Respondents in all cadres had substantial experience working in IDSR/EWARN. All respondents performed the various IDSR/EWARN tasks and duties as expected. There was adequate knowledge of case definitions and alert thresholds of priority health conditions and the timing of reporting. There was sufficient feedback using a variety of methods, although simplified feedback is needed at lower levels. Some community health workers were not familiar with IDSR/EWARN. Regular supervision from the higher levels was lacking for, the lower levels. Laboratory respondents reported a lack of standardized reporting forms, specimen transfer forms, and laboratory equipment. In terms of surveillance attributes, the IDSR/EWARN system was rated simple, flexible, with medium to high data quality, medium to high acceptability, and medium representativeness. Stability was rated high overall, and usefulness was rated high. For laboratory-based attributes, simplicity was rated high, data quality medium, and timeliness was medium to high, while stability was low.

There were a few performance gaps that were found in the evaluation. In the laboratories, confirmation of priority diseases by the laboratories needs to be strengthened. Specimen collection for suspected acute watery and bloody diarrhoea was lacking. There is a lack of standardized reporting forms, specimen transfer forms, and laboratory equipment. Health facilities need more regular supervision from the higher levels, and community health workers need more training on IDSR/EWARN. These gaps point to a lack of resources, and indeed, we identified that the IDSR/EWARN system needs more resources, particularly for communication and transport and to confirm priority diseases. Staff at all levels requested more training in IDSR/EWARN. South Sudan is implementing all components of the revised IDSR technical guidelines, given its historical use of EWARN, prioritizing rapid notification and response to epidemic-prone diseases and its early adoption of IDSR. South Sudan also relies on event-based surveillance. There are performance gaps primarily due to a lack of resources; the country is performing relatively well given its context and vast, remote areas. Several recent IDSR evaluations have indicated that training is one of the primary interventions needed for improved performance [20,21].

WHO-AFRO has started virtual training in IDSR [22]. However, focused and sustained provision of resources, including funds and equipment, is likely to lead to sustained improvement beyond training. It may be a waste of resources to conduct training for health personnel who do not have the tools to implement their training. The lack of resources for public health laboratories is an ongoing problem in several countries in the African region, and the continuing COVID-19 pandemic has worsened this [23]. The evaluation had a few limitations. The COVID-19 pandemic limited the scope of the assessment, given the restrictions on travel. Funding also limited the size of the local team to one person. Some respondents did not fill out the questionnaires, and this can limit the generalization of the findings. The evaluation was carried during the COVID-19 pandemic, and the team adapted the methods to the pandemic and triangulated the data to provide comprehensive findings. However, it is possible that the findings from the sites that did not respond could be different from those who responded, which could have introduced a selection bias. Using mixed methods (i.e. qualitative and quantitative) allowed the evaluation team to use various data sources to triangulate the evaluation results.

## Conclusion

Since the inception of IDSR in South Sudan in 2006, surveillance and response capacities have improved but remain below the IDSR program targets and the IHR (2005). All efforts should be made to maintain the surveillance performance achieved by week 16 of 2021 for both indicator and event-based surveillance and was still almost at the same level at week 32 of 2021. The information generated from the improved reporting performance should be used for public health action. For instance, rapid response teams should respond to all outbreak alerts through outbreak investigations and special epidemiological studies. Refresher training needs to be provided to all levels below the central level, particularly for the community and the health facility level. Support supervision should be strengthened for all levels. Feedback to the health facilities and communities needs to be developed and maintained
